# Continuous low-dose cyclophosphamide and methotrexate combined with celecoxib for patients with advanced cancer

**DOI:** 10.1038/bjc.2011.154

**Published:** 2011-05-17

**Authors:** O A Khan, A D Blann, M J Payne, M R Middleton, A S Protheroe, D C Talbot, M Taylor, C Han, M Patil, A L Harris

**Affiliations:** 1 University of Oxford Department of Medical Oncology, Churchill Hospital, Oxford OX3 7LJ, UK; 2 University Department of Medicine, City Hospital, Birmingham B18 7QH, UK

**Keywords:** celecoxib, low-dose chemotherapy, angiogenesis

## Abstract

**Background:**

Combined therapy of metronomic cyclophosphamide, methotrexate and high-dose celecoxib targeting angiogenesis was used in a phase II trial.

**Methods:**

Patients with advanced cancer received oral cyclophosphamide 50 mg o.d., celecoxib 400 mg b.d. and methotrexate 2.5 mg b.d. for two consecutive days each week. Response was determined every 8 weeks; toxicity was evaluated according to CTC version 2.0. Plasma markers of inflammation, coagulation and angiogenesis were measured.

**Results:**

Sixty-seven of 69 patients were evaluable for response. Twenty-three patients had stable disease (SD) after 8 weeks, but there were no objective responses to therapy. Median time to progression was 57 days. There was a low incidence of toxicities. Among plasma markers, levels of tissue factor were higher in the SD group of patients at baseline, and levels of both angiopoietin-1 and matrix metalloproteinase-9 increased in the progressive disease group only. There were no changes in other plasma markers.

**Conclusion:**

This metronomic approach has negligible activity in advanced cancer albeit with minimal toxicity. Analysis of plasma markers indicates minimal effects on endothelium in this trial. These data for this particular regimen do not support basic tenets of metronomic chemotherapy, such as the ability to overcome resistant tumours by targeting the endothelium.

Chemotherapy for the treatment of metastatic cancer mainly follows the rationale that when drugs are effective, higher doses of drugs are even more effective. Unfortunately, this has not produced the anticipated benefits for common solid tumours such as breast cancer (Stadtmauer *et al*, 2000). A re-evaluation of ‘traditional’ chemotherapy dose schedules, notably high intermittent doses, has occurred recently following the observation that low doses of chemotherapy given continuously (‘metronomic’ dosing) can be selectively toxic to proliferating endothelial cells in tumours, which tend to be resistant to standard episodic scheduling (Kerbel and Kamen, 2004). This has an impact on tumour angiogenesis and so tumours resistant to normal bolus doses of chemotherapy may respond to low chronic doses of cytotoxics and with different classes of anti-cancer drugs, such as the combination of low-dose vinblastine chemotherapy and a vascular endothelial growth factor (VEGF) receptor 2 antibody (Klement *et al*, 2000).

Cyclooxygenase (COX) catalyses reactions needed for the formation of prostaglandins from arachidonic acid. Cyclooxygenase-1 is constitutively expressed and maintains normal cellular physiological functions such as platelet aggregation and gastric cytoprotection (Needleman *et al*, 1986). Isoenzyme COX-2 expression is upregulated in inflammation, human tumour neovasculature and in neoplastic cells present in human colon, gastric, breast, prostate, endometrial, skin and lung cancer biopsy tissue (Masferrer *et al*, 2000; Gately and Kerbel, 2003). There is increasing evidence that COX-2 modulates angiogenesis by augmenting the release of angiogenic peptides such as VEGF, basic fibroblast growth factor (bFGF) and nitric oxide (Gately and Kerbel, 2003). Inhibition of COX-2 by non-steroidal anti-inflammatory drugs (NSAIDs) results in reduced angiogenesis and downregulation of proangiogenic factors, such as VEGF and bFGF. Indeed, the incidence of colorectal cancer in patients taking NSAIDs is significantly lower than in those who are not (Dubois *et al*, 1998). A recent study showed that tumour growth was suppressed by inhibiting angiogenesis with a COX-2 inhibitor both *in vitro* and *in vivo* and this was not seen in normal endothelial cells (Muraki *et al*, 2011). There was also a significant suppression of CD133+/vascular endothelial growth factor receptor cells in the circulation by COX-2 inhibition.

Clinically, selective COX-2 inhibition is a more attractive approach due to the lower risk of gastrointestinal toxicity than non-selective NSAIDs. Celecoxib is a selective COX-2 inhibitor (Penning *et al*, 1997) and is the first COX-2 inhibitor that was given FDA approval and was approved in the UK in 2000, although two trials in patients with adenomatous polyps have been halted prematurely over concerns about its cardiovascular safety (Solomon *et al*, 2005). A report of the VICTOR study, which was a phase III placebo-controlled trial of rofecoxib in non-metastatic colorectal cancer patients, has demonstrated no effect on overall survival (OS) when compared with placebo, but there is a suggestion of a protective effect against recurrence, though this was not statistically significant (Midgley *et al*, 2010).

Combining low-dose chemotherapy with anti-angiogenic drugs is a rational approach to treating drug-resistant cancers, as it targets both endothelial cells and mechanisms of angiogenesis. Accordingly, we hypothesised that a combination of low-dose chemotherapy with celecoxib could be effective in patients with advanced cancer who were heavily pre-treated with standard chemotherapy and consequently likely to have drug-resistant tumours.

We tested our hypothesis in four different patient groups: breast cancer, gastrointestinal cancer, melanoma and other cancers (ovarian, prostate, renal and unknown primary). The main purpose of the trial was to assess efficacy of the combination in these patients as well as to provide an assessment of safety. Additionally, we evaluated the potential for plasma proteins to act as surrogate angiogenic markers that could be used to monitor treatment activity or be related to achievement of disease stabilisation. These were based on literature reports defining the mechanisms of action in preclinical studies, for example downregulation of anti-angiogenic mechanisms (thrombospondin, TSP) (Lawler, 2002; Bocci *et al*, 2003), expression of key angiogenic mediators and cytokines (VEGF (Senger *et al*, 1993), angiopoietins-1 and -2 (ANG-1 and ANG-2) (Davis *et al*, 1996; Maisonpierre *et al*, 1997) and interleukin-6 (IL-6)) (Cohen *et al*, 1996), tissue factor (TF) (Rickles *et al*, 2003), apoptosis markers (M30) (Gately and Kerbel, 2003) or proteins expressed by endothelial cells (Von Willebrand factor(VWF), soluble E-selectin (sEsel)) (Gehan, 1961; Kerbel and Kamen, 2004) and involved in vascular remodelling (matrix metalloproteinase-9 (MMP-9)) (Bergers *et al*, 2000).

## Materials And Methods

### Patient selection

Adult patients with histologically or cytologically confirmed cancer and evaluable lesions were considered for the study at two clinical centres. Inclusion criteria included WHO performance status (PS) 0–2 and adequate baseline organ function (absolute neutrophil count ⩾1.5 × 10^9^ per l, platelet count ⩾100 × 10^9^ per l, bilirubin ⩽1.5 times upper limit of normal, transaminases <5 times upper limit of normal and creatinine ⩽150 *μ*M). Patients could not have had radiotherapy, chemotherapy or immunotherapy within the previous 4 weeks (or 6 weeks for nitrosoureas or mitomycin-C). Patients were also excluded if they had active symptomatic disease of the central nervous system, uncontrolled non-malignant disease or a history of gastrointestinal bleeding in the preceding 6 months. A history of allergy to any of the trials drugs was also an exclusion criterion. Patients who had received radiotherapy were included if their assessable disease was outside the radiation field. Written informed consent was obtained from all patients before enrolment. The study was conducted with the approval of the Oxford Research Ethics Committee.

### Treatment

Patients received treatment with cyclophosphamide 50 mg o.d. and celecoxib 400 mg b.d. for 7 days each week, with methotrexate 2.5 mg b.d. for two consecutive days each week. The doses of cytotoxic drugs were selected based on a report of another trial, which utilised the same combination (Colleoni *et al*, 2002). The dose of celecoxib selected is the standard therapeutic dose. All the drugs were administered PO. Treatment was continued until grade 3 or 4 toxicity and if and when this improved to a grade 2 or better, it was continued with a 25% reduction in the dose of cyclophosphamide and methotrexate. Treatment was stopped altogether if a further grade 3 toxicity occurred. Treatment was continued until progressive disease (PD), patient refusal of treatment, investigator decision or significant intercurrent illness. Palliative and supportive care for disease-related symptoms was offered to all participants, but the use of aspirin and non-steroidal anti-inflammatory drugs was not permitted under the study protocol.

### Evaluations

Toxicity was graded according to the NCI Common Toxicity Criteria version 2. For grades 1 and 2 events treatment was continued. Dose adjustments were made in the event of grade 3 or 4 toxicities. Treatment was discontinued until the toxicity resolved to grade 2 or better and was then restarted with a 25% reduction in the doses of cyclophosphamide and methotrexate. If a further grade 3 or 4 toxicity occurred, then treatment was stopped altogether. Delays of over 2 weeks also led to withdrawal from the trial.

Tumour response was assessed according to WHO criteria, with radiological investigations repeated every 8 weeks (Therasse *et al*, 2000). Time to disease progression was calculated from the date of starting treatment until the date upon which disease progression was first recorded. Stable disease (SD) duration of 3 months was considered a response. Survival was determined from the first day of treatment until the date of death.

### Surrogate markers of tumour angiogenesis

Surrogate markers measured in plasma were MMP-9, VEGF, IL-6, thromospondin (TSP), apoptosis marker (M30), TF, ANG-1, ANG-2, VWF and sEsel (commercial ELISAs, e.g. Dako-Patts, Ely, UK and R&D Systems, Abingdon, UK). Intra-assay coefficients of variation were <5% and inter-assay coefficients were <10%. Markers were measured in plasma at baseline, after weeks 4 and 8 of therapy.

### Statistical methods

The sample size was selected on the basis that a minimum of 14 patients would ensure that the standard error of the observed response rate was ⩽0.1 and permit a satisfactory estimate of response rate, but incorporated an early stopping rule according to the method of Gehan (1961), with the response rate of interest set at 20%. This was for each tumour type, but once four main ones were accrued, the study stopped because of slow recruitment in other tumour types. Numbers exceed 14, where 1 or more cases with SD were observed in the in first 14 cases. Descriptive statistics were generated for efficacy, toxicity and pharmacodynamic end points. The median time to progression (TTP) was estimated using Kaplan–Meier survival curves (STATA-1). Angiogenic marker data were subjected to the Anderson–Darling distribution test to direct parametric/non-parametric mode of testing, and is presented as mean and s.d. (when data normally distributed) or median and inter-quartile range (non-normally distributed). The *χ*^2^ test was used for categorical data; Spearman's method was used for correlations. Data between groups were analysed by *t*-test or the Mann–Whitney *U-*test. Data at three time points were analysed by Friedman's two-way repeated measures analysis of variance. The log-rank test was used to compare survival between patients with progressive and SD. Significance was assumed if *P*<0.05. All analyses were performed on Minitab release 15.

## Results

### Patient characteristics

Seventy-four patients with advanced cancer were enrolled, of whom five patients were not well enough to proceed to treatment. All patients had PD at baseline. Sixty-nine patients completed at least 1 week of celecoxib (median duration that patients received treatment was 10 weeks: range: 1–48 weeks). Fourteen patients were on study for <8 weeks, 34 were on study between 8 and 16 weeks, 11 were on study between 16 and 24 weeks and 10 patients were on study for over 24 weeks. The baseline and demographic characteristics for these 69 patients are shown in Table 1. Most patients had a PS of 0 or 1. The median age was 60 (range: 30–82). The majority of patients had multiple sites of metastatic disease of six different primary origins with the most common disease spread to liver (39%), lungs (35%) and bone (17%). All patients were heavily pre-treated with a median of two previous chemotherapy regimens (range: 1–7) as well as other therapies (Table 1). All 69 patients were assessable for safety. Two patients were excluded from efficacy evaluation with insufficient data. The first of these patients had melanoma and was taken off study on the eighth day. The second of these had prostate cancer and was considered non-eligible as he was enrolled in the study without the knowledge that he had life-long asthma, which is an exclusion criterion. He was admitted to hospital with breathlessness and symptoms of a chest infection, the investigators become aware of his asthma and the patient was withdrawn from the study.

### Toxicity

As expected, the incidence of grade 3 or 4 haematological toxicities was low and similarly, other grade 3 or 4 toxicities were rare (Table 2). One patient with renal cell cancer presented with agitation and acute onset confusion after 6 weeks on study. This resolved within 24 h and CT of the head was normal. He was diagnosed with a transient ischaemic attack.

### Dose administration and dose intensity

Celecoxib dose was maintained for all patients on the trial, in keeping with there being no protocol-defined criterion for its reduction. Methotrexate dose was reduced by 25% for five patients and cyclophosphamide dose was reduced by 25% for four patients. There was thus one patient in whom the dose of cyclophosphamide was not reduced as per protocol.

### Tumour response

**Figure 1:**
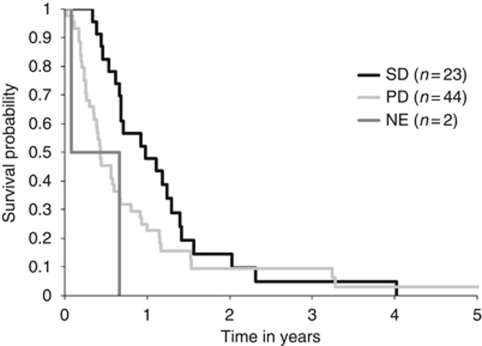
OS shown by treatment outcome. SD=stable disease; PD=progressive disease; NE=non-evaluable.

There were no objective responses, but 23 of 67 patients (34.3%) evaluable had SD at 12 weeks. The other 44 patients (65.7%) progressed. Best response by site of disease is shown in Table 3. The median TTP was 57 days (range: 2–338). Median OS was 226 days (range: 9–846). The TTP of breast cancer patients was longer than in the other groups (82 days) and the median OS of melanoma patients was shorter than in the other groups (139 days). Survival by response is shown in Figure 1. Patients with SD survived longer than those with PD, although this was not quite statistically significant (*P*=0.06).

### Plasma markers

Results were available for 41 patients (breast cancer: 9, gastrointestinal cancer: 9, renal carcinoma: 2, melanoma: 11, ovarian cancer: 3 and prostate cancer: 7). Samples were only taken from one of the research sites. Table 4 shows changes in the angiogenic markers at the three time points for those in each of the two groups. In cross-sectional analysis, levels of ANG-1 were significantly lower in the PD group compared with the SD group at baseline. Levels of MMP-9 were higher in the PD group compared with the SD groups at the third time point only. Levels of TF were higher in the SD group at all three time points. In serial analysis, levels of both ANG-1 and MMP-9 increased over the three time points in the PD group only. There were no other statistically significant changes.

## Discussion

This study was designed to assess tumour response to low-dose continuous (metronomic) chemotherapy with high-dose celecoxib in patients with advanced, metastatic cancer and to assess the effects of the combination on surrogate markers of tumour angiogenesis and their relation to disease stabilisation. The combination of cyclophosphamide and methotrexate with celecoxib is based on the postulated importance of angiogenesis in tumour growth, the *in vitro* and *in vivo* anti-angiogenic and anti-neoplastic properties of celecoxib and early clinical experience of metronomic chemotherapy in breast and prostate cancer when standard maximally tolerated dose of chemotherapy has failed. Combination therapy resulted in over 4 month's disease stabilisation in a minority (34.3%) of patients and was well tolerated with a low incidence of haematological and non-haematological toxicities. No excess cardiovascular toxicity was observed, although one patient experienced a grade 3 ischaemic attack. Patients were enrolled with high levels of transaminases to include patients with advanced disease, including liver metastases in which a metronomic approach may have been a more suitable option than standard chemotherapy due to lower toxicities. Indeed, there were no significant grades 3 or 4 hepatic toxicities or discontinuations due to hepatic toxicity despite both methotrexate and celecoxib being potentially hepatotoxic drugs.

Clinical experience with metronomic chemotherapy and COX-2 inhibitors is limited, but there have been a number of recent reports suggesting some activity. Colleoni *et al* (2002, 2006) reported results with low-dose chemotherapy in patients with metastatic breast cancer in two trials. In the earlier trial, clinical benefit rate (CBR: defined as CR+PR+SD for 24 weeks) was 31.7% and in the second, this was even higher at 41.8%. However, in the first study, the objective response rate (ORR: defined as patients demonstrating complete response or partial response on two separate measurements at least 4 weeks apart) was 19%. Importantly, almost one-fifth of the patients had not received previous treatment for metastatic disease. In the second, the ORR was 16.3%, but almost 40% of patients had been previously untreated for metastatic disease; in pre-treated patients, the ORR was only 11.8%. In contrast to our study, the PS of patients was mostly 0 or 1 in both these studies. Toxicity was generally mild in both trials.

A phase II trial involving treatment with low-dose oral cyclophosphamide, weekly vinblastine and rofecoxib in 47 patients with advanced solid tumours reported a CBR of 30% with minimal toxicity (Young *et al*, 2006). The ORR was only 13% in a group of mostly good PS patients. Of the responders, three patients had Hodgkin lymphoma and two others had been untreated for metastatic disease.

In a small trial, 35 patients were treated with high-dose celecoxib and low-dose cyclophosphamide in relapsed or refractory non-Hodgkin's lymphoma. The ORR was 37% (Buckstein *et al*, 2006). Other clinical studies of these drugs in different cancers have reported varying degrees of success (Needleman *et al*, 1986; Masferrer *et al*, 2000; Pasquier *et al*, 2010; Muraki *et al*, 2011), although many have not demonstrated significant efficacy (Stempak *et al*, 2006; Kesari *et al*, 2007; Steinbild *et al*, 2007; Stockhammer *et al*, 2010).

There have been some clinical trials that have reported encouraging results with metronomic chemotherapy combined with other anti-angiogenic drugs. One of these was in breast cancer, where 46 patients with advanced disease were treated with metronomic oral capecitabine and cyclophosphamide plus bevacizumab (Dellapasqua *et al*, 2008). Objective response rate was 48% with a CBR of 68% and mild toxicities. The results of this trial were considered to be of sufficient importance that a phase III trial of this combination is being investigated in Europe. In heavily pre-treated ovarian cancer, 70 patients were treated with oral cyclophospamide and bevacizumab and the ORR was 24% with a CBR of 56% (Garcia *et al*, 2008).

Recently, there have been some reports that have challenged the concept of the anti-angiogenic effects of celecoxib (Xu *et al*, 2011). The effects of celecoxib in glioma cell lines and xenografts included induction of VEGF expression at a similar level to that induced by hypoxia and formation of new blood vessels *in vivo*. The authors suggested that cytotoxicity of celecoxib might be due to other effects, such as those on apoptosis rather than on angiogenesis.

Angiogenic therapies often do not induce tumour regression and, therefore, identification and validation of soluble markers of anti-angiogenic activity is essential for successful integration of anti-angiogenic therapy into clinical practice. We evaluated changes in soluble markers of angiogenesis in an attempt to correlate changes in these variables with clinical and radiological response. Of these markers, the only significant difference between patients with SD and PD at baseline was ANG-1 and TF, both higher by factors of 5 and 6, respectively, in those whose disease was stable. Whether this implies a more sensitive vascular bed is not possible to assess. Serial studies showed that levels of MMP-9 increased in line with disease progression, but were stable in those whose disease was stable. In other serial analysis, levels of ANG-1 also increased over the three time points in the PD group only. Surprisingly, levels of other angiogenic cytokines (ANG-2 and VEGF) failed either to differentiate stable from PD, or to be influenced by the treatments. Pre-treatment blood levels of VEGF have been tested in many studies and in general, elevated levels are indicative of poorer prognosis, but do not predict response to anti-angiogenic drugs (Sessa *et al*, 2008).

Exploratory analysis of outcome by response showed clearly that those with SD had longer survival (Figure 1). As this is a phase II trial, it could be due to intrinsically slower progression of one group of patients, but this could also reflect a difference in the biology and response to therapy, which can only be resolved by randomisation. In all, 7 out of the 10 patients who had long control of disease (>24 weeks) had either breast or colorectal cancer, suggesting that the reason for SD may be due to underlying histology. If those patients who had disease stabilisation for longer than 16 weeks are also included, then 11 out of 21 patients had either breast or colon cancer and a further 2 patients had kidney cancer, all of which are tumour histotypes that may have a more indolent course. Additionally, there was no difference in how heavily pre-treated the patients were when the patients with SD were compared with those with PD, thus ruling out the possibility that benefit might be greater in less pre-treated patients.

Our data do not support one of the key postulated mechanisms of action of metronomic therapy, that is suppression of TSP, and do not show that VEGF is a good marker for angiogenesis in this setting. A number of other trials using metronomic chemotherapy with or without celecoxib have also reported mixed results when assessing biomarkers of angiogenic activity with most reporting negative findings. For example, in a trial of low-dose metronomic vinblastine and cyclophosphamide with celecoxib for paediatric solid tumours, VEGF, bFGF, sVCAM-1, sICAM-1, endostatin and TSP were measured (Stempak *et al*, 2006). The results were highly variable and no statistically significant relationship between them and disease progression or maintenance of SD was observed which is likely to be a reflection of small sample size.

In the trial of low-dose chemotherapy in breast cancer conducted by Colleoni *et al* (2002), there was a significant drop in median VEGF levels comparing baseline to 2 months. There was no difference in median reductions between responders and non-responders, but the reduction was significant only in responders. Overall, the conclusion was that there was no evidence that baseline serum VEGF is associated with predicting response and relative change in serum VEGF from baseline to 2 months was not predictive of response. In a more recent trial in metastatic breast cancer, there was a 30% reduction in serum VEGF after 2 months in patients with CR or PR and a 14% reduction in patients with SD. There was no significant reduction in patients with PD (Colleoni *et al*, 2006). In a phase II trial of metronomic etoposide and cyclophosphamide in combination with daily thalidomide and celecoxib in adults with recurrent malignant gliomas, there was minimal anti-tumour activity and there were no statistically significant differences between responders and non-responders in changes in serum or urine levels of bFGF or VEGF (Kesari *et al*, 2007).

There is an inconsistent relationship between soluble markers of angiogenesis and response to anti-angiogenic therapy and the practical utility of using drug-induced increases in circulating factors as surrogate biomarkers remains to be demonstrated (Sessa *et al*, 2008). The lack of normal reference ranges makes it difficult to interpret results. The most specific endothelial markers, VWF and soluble sEsel were, like VEGF and ANG-2, unable to differentiate disease outcome at baseline or response to treatment. However, no changes in these endothelial markers imply lack of damage/dysfunction, suggesting that the therapy is not sufficiently cytotoxic to this organ. In contrast, increased VWF after therapy such as steroids and cisplatin is taken as a reflection of vascular damage (Angles-Cano *et al*, 1979; Licciardello *et al*, 1985).

Recently, there has been increasing evidence that tumour endothelial cells may harbour tumour specific genetic abnormalities and, therefore, may acquire drug resistance (Pasquier *et al*, 2010). Consequently, detailed pharmacogenetic studies on tumour endothelial cells will be needed in future trials of metronomic chemotherapy. There has also been increasing evidence to support additional mechanisms of action of metronomic chemotherapy beyond that of the anti-angiogenic paradigm. These include inhibitory effects on regulatory T cells leading to an increased anti-tumour immune response, induction of tumour dormancy and direct effects on cancer cells and cancer stem cells (Pasquier *et al*, 2010).

In conclusion, this combination of daily cyclophosphamide, weekly methotrexate given concurrently with daily celecoxib provided little anticancer activity in a variety of heavily pre-treated patients with advanced solid tumours. The data with this particular combination do not support the basic tenets of metronomic chemotherapy, such as the ability to overcome resistant tumours by targeting the vessels. There has been some evidence recently that disputes the anti-angiogenic effects of COX-2 inhibitors, which might explain why this study among others have seen only minor or no responses with little or no effects on vascular markers of angiogenesis. There may, therefore, be more merit in pursuing combination metronomic therapy trials with drugs with a more established anti-angiogenic mechanism of action.

## Figures and Tables

**Table 1:** Clinical and demographic details of the patients and details of previous treatments

Male	23
Female	46
Age (range)	59.5 (30.2–82.2)
	
*Performance status (ECOG)*	
0	19
1	33
2	10
Unk	7
	
*Previous treatment*	
Surgery	52
Radiotherapy	31
Hormonal/biological	35
Chemotherapy	50
	
Prior chemotherapy	
Median no prior regimens (range)	2 (1–7)

Abbreviation: ECOG=Eastern Cooperative Oncology Group.

**Table 2:** Number of patients with grade 3/4 adverse events considered to be related to study treatment

**Adverse event**	**Grade 3**	**Grade 4**
Neutropaenia	1	0
Lymphopaenia	22	0
Thrombocytopaenia	1	0
Anaemia	2	1
Fatigue	11	0
Neurological	6	0
Lethargy	3	0
Diarrhoea	3	0
Dyspnoea	1	0
Vomiting	1	1
Malaise	1	0
Indigestion	1	0
Ischaemic attack	1	0

**Table 3:** Best response per patient and site of disease

**Best response site**	**Not evaluable**	**PD**	**SD**	**Total**
Breast	0	10	5	15
GI	0	10	7	17
Renal	0	5	2	7
Melanoma	1	10	4	15
Ovary	0	1	2	3
Prostate	1	5	3	9
Unknown primary	0	3	0	3
Total	2	44	23	69

Abbreviations: GI=gastrointestinal; PD=progressive disease; SD=stable disease.

**Table 4:** Plasma angiogenic marker data

**Marker**	**Time point**	**Result (PD)**	**Result (s.d.)**	***P* (between groups)**
ANG-1	1	1.0 (0.3–6.0)	5.0 (1.5–28.0)	**0.018**
	2	3.0 (0.5–6.5)*	4.2 (1.2–10.7)	0.297
	3	3.0 (0.4–9.0)^*^	7.0 (2.8–34.5)	0.079
ANG-2	1	7.0 (4.0–9.7)	7.5 (5.0–15.5)	0.264
	2	6.0 (4.7–10.0)	13.0 (10.5–18.5)	0.224
	3	7.0 (5.2–10.7)	8.0 (5.5–18.0)	0.651
IL-6	1	13.0 (10.5–18.5)	19.0 (10.0–25.0)	0.194
	2	15.0 (10.0–22.5)	15.5 (8.5–22.3)	0.947
	3	15.5 (5.0–19.5)	15.0 (6.5–57.5)	0.995
M30	1	41.0 (30.0–175.0)	53.0 (30.0–80.0)	0.769
	2	41.0 (30.0–147.5)	63.0 (30.0–117.0)	0.841
	3	40.0 (30.0–140.0)	50.0 ( 30.0–177.0)	0.995
MMP-9	1	60 (45–71)	50 (40–62)	0.088
	2	70 (56–72)^*^	60 (47–69)	0.095
	3	72 (60–74)^*^	55 (43–61)	**0.001**
sEsel	1	92.5 (62.5–107.5)	65.0 (52.5–132.5)	0.533
	2	92.5 (70.0–125.0)	75.0 (52.5–115.0)	0.321
	3	90.0 (70.0–117.0)	70.0 (55.0–92.5)	0.193
TF	1	10.0 (5.3–87.5)	60.0 (21.0–235.0)	**0.045**
	2	13.0 (8.0–79.0)	80.0 (41.0–10180)	**0.027**
	3	19.0 (10.0–64.0)	140.0 (50.0–10180)	**0.012**
TSP	1	200 (4.5–500.0)	60 (4.4–300)	0.727
	2	250 (6.2–500.0)	50 (5.8–287)	0.209
	3	250 (112–587)	250 (250–400)	0.671
VEGF	1	450 (125–1900)	500 (100–800	0.989
	2	250 (113–1450)	320 (250–750)	0.378
	3	280 (200–4500)	400 (130–75000)	0.630
VWF	1	115 (99–144)	112 (103–141)	0.856
	2	120 (100–138)	130 (103–146)	0.621
	3	112 (93–128)	103 (84–156)	0.977

Abbreviations: ANG-1=angiopoietin-1; ANG-2=angiopoietin-2; IL-6=interleukin-6; MMP-9=matrix metalloproteinase-9; PD=progressive disease; sEsel=E-selectin; TF=tissue factor; TSP=thrombospondin; VEGF=vascular endothelial growth factor; VWF=Von Willebrand factor.

1, 2 and 3 refer to visit number. Data presented as median and interquartile range. *P-*value between groups at each time point by the Mann–Whitney *U-*test. Statistically significantly different *P-*values are in bold. ^*^P<0.05.
