# Continuous low-dose cyclophosphamide and methotrexate combined with celecoxib for patients with advanced cancer

**DOI:** 10.1038/bjc.2011.411

**Published:** 2011-10-11

**Authors:** O A Khan, A D Blann, M J Payne, M R Middleton, A S Protheroe, D C Talbot, M Taylor, O Kirichek, C Han, M Patil, A L Harris

**Correction to**: *British Journal of Cancer* (2011) **104**, 1822–1827; doi:10.1038/bjc.2011.154

When originally published earlier this year in Volume 104, the authorship of this paper was incorrect. O Kirichek who is based, along with several of the other authors at Department of Medical Oncology, University of Oxford, Churchill Hospital, Oxford, UK, was omitted from the author list.

The full author listing is now shown, above. The authors would like to apologise for this mistake.

